# Effects of a 6-month exercise programme on disease activity, physical and functional parameters in patients with ankylosing spondylitis: Randomised controlled trial

**DOI:** 10.4102/sajp.v77i1.1546

**Published:** 2021-06-29

**Authors:** Kim Nolte, Dina C. Janse van Rensburg, Lizelle Fletcher

**Affiliations:** 1 Department of Physiology, Division of Biokinetics and Sport Science, Faculty of Health Sciences, School of Medicine, University of Pretoria, Pretoria, South Africa; 2 Section Sport Medicine, Faculty of Health Sciences, University of Pretoria, Pretoria, South Africa; 3 Department of Statistics, Faculty of Natural and Agricultural Sciences, University of Pretoria, Pretoria, South Africa

**Keywords:** ankylosing spondylitis, arthritis, aquatics, exercise therapy, rehabilitation

## Abstract

**Background:**

Exercise forms an important component in the management of ankylosing spondylitis (AS). The objective of our study was to determine the effects of a 6-month swimming, land-based stretching, strengthening and breathing exercise intervention in AS patients.

**Methods/design:**

A total of 29 (14 females, 15 males) patients diagnosed with AS according to the Modified New York Criteria participated in our study. Participants were randomly assigned to an exercise group (ASE) (16 participants) and a control group (ASC) (13 participants). Erythrocyte sedimentation rate and C-reactive protein (CRP); anthropometric measurements; pulmonary function; aerobic capacity; balance; Bath AS Metrology Index; Bath AS Disease Activity Index and the Bath AS Functional Index were assessed.

**Discussion:**

Erythrocyte sedimentation rate and CRP did not change notably within or between the groups from pre- to post-intervention. The between group analyses of the physical assessments favoured the ASE with observable improvements in chest expansion (*p* = 0.002), forced expiration volume (*p* = 0.012), absolute (*p* = 0.017) and relative (*p* = 0.003) maximal oxygen consumption, absolute (*p* = 0.028) and relative (*p* = 0.001) physical work capacity. Within the ASE, there is statistical evidence of improvements in 11 of the 19 physical variables. Within the ASC, five of the physical variables deteriorated substantially (*p* between 0.004 and 0.037) and only balance overall stability on the right improved (*p* = 0.016). The three functional assessments in the ASE improved (*p* < 0.007) compared with the ASC post-intervention. A 6-month combined exercise programme may improve physical and functional capacity of AS patients.

**Conclusion:**

A multimodal exercise intervention may be useful in the management of ankylosing spondylitis.

**Clinical implications:**

An unsupervised well -explained exercise programme combining swimming, land-based strengthening and stretching exercises and breathing exercises may be an option for patients with ankylosing spondylitis.

**Trial registration:**

Registration not undertaken at the time of writing.

## Introduction

Ankylosing spondylitis (AS) is a chronic, systemic, rheumatic disease that is a prototype of seronegative spondyloarthropathies and characterised by inflammation, primarily of the spine (Ince, Sarpel & Durgun 2006). The main clinical characteristics of AS are reduced physical activity, pain, stiffness, sleep disturbances, decreased spinal mobility and psychological consequences such as depression (Lim, Lim & Lee 2005). Ankylosing spondylitis management strategies should focus on reducing disease activity, improving lumbar spinal mobility and functional status (Özdemir 2011). Treatment of AS usually includes the use of anti-inflammatory drugs to reduce pain and stiffness and disease modifying drugs to try to stop or prevent disease progression. Patients are also advised to exercise to maintain mobility of the spine and peripheral joints (Hidding et al. 1993). A growing body of research reveals that exercise is as crucial as drug treatment in the management of AS (Dougados & Van der Heijde 2002; Heikkilä et al. 2000; Nolte & Janse van Rensburg 2001). Functional limitations in early AS may be closely related to symptoms, whereas functional limitations in long-standing AS may be more closely related to cumulative factors leading to spinal fusion and long-term structural damage (Ward et al. 2005).

In AS, the main objectives of exercise therapy are prevention and delay of stiffness, pain control, postural re-education and improvement of functional and cardiorespiratory function (O’Dwyer, O’Shea & Wilson 2014). Considering the numerous structures and functions that are influenced by the disease, exercise prescription for AS patients is not simple and should probably include various modalities and types of exercises. The knowledge of aquatic exercise in AS is still limited (Zão & Canista 2017). However, it appears as if water exercise may have additive beneficial effect on pain, mobility, functionality and quality of life (Fernández et al. 2014; Karapolat et al. 2009).

Few randomised controlled studies have examined the effects of long duration (more than 3 months) exercise programmes specifically combining swimming and land-based stretching, strengthening and breathing exercises in AS patients. It has also been recommended that the quality of interventions in exercise trials for patients with AS can be improved and that future trials should focus on measuring and reporting physiological responses and adherence to exercise intervention (Daginfrud et al. 2011). A recent meta-analysis of randomised controlled trials on the effects of exercise programmes in AS patients concluded that further studies are needed to investigate the effects of specific exercise interventions on pain, quality of life, function and disease activity (Hu et al. 2020). Our study aimed to determine the effects of a 6-month swimming, land-based stretching, strengthening and breathing exercise intervention on disease activity, physical- and functional-parameters in patients with AS by comparing an exercise group (ASE) with a control group (ASC).

## Methods

A total of 29 patients (14 females, 15 males) diagnosed with AS according to the Modified New York Criteria, between the ages of 18–70 years were recruited for our study. Eligible patients were referred by rheumatologists in private and public settings. Ankylosing spondylitis is a rare disease with the reported prevalence in Africa at 7.4 per 10 000 (Dean et al. 2014). Only patients in close proximity to the testing facility could be recruited, and were eligible to participate if they could communicate in English, knew how to swim, were not currently participating in a structured exercise programme and were on stable medication for the last 3 months. Ankylosing spondylitis medication (dosage and type) was not changed or adjusted during the period they were involved in our study. In case of disease exacerbation, participants were allowed to use oral analgesics or topical anti-inflammatory ointments. Exclusion criteria were an inability or unwillingness to participate in an exercise programme and comorbidity of heart, lung, liver or kidney disease as confirmed by the rheumatologist. Participants completed and signed informed consent prior to initiation of our study. Allocation to groups was not concealed and participants were randomly divided into two groups by the trial coordinator during their baseline assessment using a random number table: an ASE (16 participants [nine females, seven males]) and an ASC (13 participants [seven females, six males]). The participants and biokineticist (responsible for the assessments and intervention) were not blinded to the group allocation.

### Assessments

Participants were assessed three times during the course of our study (0 months, 3 months and 6 months). Assessments at the start (0 months) and 6 months are reported on. Assessments included inflammatory blood markers, as well as physical and functional assessments. The inflammatory blood markers comprised of erythrocyte sedimentation rate (ESR) and C-reactive protein (CRP). The physical assessments included anthropometric measurements to determine chest wall expansion (CWE) and body composition. Anthropometric measurements included: body mass (kg), stature (cm), four skin folds (triceps, biceps, subscapular and suprailiac), two bony breadth measurements (humerus and femur) and four circumference measurements (biceps contracted, standing calf, maximum chest and minimum chest). Anthropometric measurements were taken according to the International Society for the Advancement of Kinanthropometry (ISAK) guidelines (Marfell-Jones & Olds 2006). Body mass index (BMI), percentage body fat (PBF) and lean body mass (LBM) were determined. Pulmonary function was assessed using a portable spirometer (Spiroflow 2000, USA). Standardised procedures conforming to the Series ‘American Thoracic Society (ATS) / European Respiratory Society (ERS) Task Force: Standardisation of Lung Function Testing, Number 2: Standardisation of Spirometry’ were used (Miller et al. 2005). The forced vital capacity (FVC, mL), the forced expiration volume at the first second (FEV1, mL) and FEV1/FVC (%) were recorded. Aerobic capacity was assessed using an incremental bicycle ergometer test.The test was started with an initial load of 25 W at a cadence of 60–70 revolutions per minute, with an increment increase of 25 W until exhaustion. Physical work capacity (PWC) was recorded (w and w/kg) and the Astrand-Rhyming protocol was used to calculate the estimated maximal oxygen consumption (VO_2max_) (mL/kg/min) of each participant. Balance was assessed on the Biodex balance SD^TM^ (Biodex, United Kingdom). The athletic single leg stability test was used to determine unilateral balance. Three familiarisation trials were allowed. Overall stability (right and left) was recorded. Spinal flexibility was determined using the Bath Ankylosing Spondylitis Metrology Index (BASMI). The BASMI is composed of five measurements: cervical rotation, tragus-to-wall distance, lumbar lateral flexion, modified Schober’s distance and intermalleolar distance (Lim et al. 2005). Each measurement indicates either 0 (mild disease involvement), 1 (moderate disease involvement) or 2 (severe disease involvement) points, resulting in a total BASMI score 0–10. Physical assessments were completed by a qualified biokineticist with an ISAK level 1 accreditation.

Participants were required to complete two questionnaires in English; the Bath Ankylosing Spondylitis Disease Activity Index (BASDAI) and the Bath Ankylosing Spondylitis Functional Index (BASFI). The BASDAI is a self-administered questionnaire consisting of six questions relating to the five major symptoms of fatigue, spinal pain, joint pain or swelling, areas of localised tenderness and morning stiffness measured in terms of severity and duration. The overall BASDAI score ranges from zero to 10, with higher scores indicating more severe symptoms (Lim et al. 2005). The BASFI consists of eight questions on daily activities and two additional questions that assess patients’ ability to cope with everyday life. The mean of the 10 scales gives the BASFI score (0–10), with higher scores indicating more severe impairment (Karatepe et al. 2005). The BASMI, BASFI and BASDAI are validated instruments to assess spinal flexibility, disease activity and functional index in patients with AS and have been used in numerous studies (O’Dwyeret al. 2014).

### Intervention

Participants in the ASC were instructed to continue with their current lifestyle and were educated on a suitable exercise programme at the conclusion of our study. Participants in the ASE each received an individual session with the biokineticist. She went through the exercise programme to ensure that they understood and could perform the exercises properly. They were instructed to exercise three times a week and were required to keep an exercise logbook in order to determine compliance with the programme. Participants were not supervised during their training but could use the pool and exercise facilities of the institution. The exercise difficulty was increased each month or 4-week period to ensure adequate progression. The swimming programme initially consisted of 10 min – 12 min of swimming (participant could select swimming stroke of choice and could rest as required during the swimming training session). At the end of the 6-month period, participants were required to swim for 30 min as continuously as possible (preferably freestyle swimming stroke at a rate of perceived exertion of 13–15). In addition to the swimming, participants were required to complete land-based exercises that included postural correction exercises, strengthening exercises, stretches and pulmonary or breathing exercises. Participants were given an exercise therapy ball at the initiation of our study as some of the land-based exercises required the use of such a ball. Land-based exercises also increased in difficulty each month (4-week period). An example of the land-based exercises is presented (Table 1).

**TABLE 1:** Land-based exercises.

Exercise	Month 1	Month 2	Month 3	Exercise	Month 4	Month 5	Month 6
**Postural correction and strengthening exercises**							
Opposite arm and leg lift	10–15 reps (R & L) 1–2 sets Performed on hands and feet	10–15 reps (R & L) 2–3 sets Performed on hands and feet	10–15 reps (R & L) 2–3 sets Performed on exercise ball	Opposite arm and leg lift	10–15 reps (R & L) 1–2 sets Performed prone on the floor	10–15 reps (R & L) 2–3 sets Performed prone on the floor	10–15 reps (R & L) 2–3 sets Performed prone on the floor Dumbbells
Standing shoulder rolls (backwards) with or without resistance	10–15 reps 1–2 sets	10–15 reps 2–3 sets	15–20 reps 2–3 sets Dumbbells	Reverse flies	10–15 reps 1–2 sets Performed standing	10–15 reps 2–3 sets Performed on exercise ball	10–15 reps 2–3 sets Performed on exercise ball Dumbbells
Hip extension (hip lift)	15–20 reps 1–2 sets	15–20 reps 2–3 sets	10–15 reps (R & L) 2–3 sets Performed on one leg	Hip extension (hip lift)	15–20 reps 2–3 sets Performed with feet on exercise ball	15–20 reps 3 sets Performed with feet on exercise ball. Hold last rep for 20 s – 30 s	15–20 reps R & L 2–3 sets Performed with one foot on exercise ball. Hold last rep for 10 s –15 s
Front plank	Time held: 30 s – 60 s 1–2 reps Performed on elbows and feet	Time held: 30 s – 60 s 2–3 reps Performed on hands and feet	Time held: 60 s 2–3 reps Performed on hands and feet	Front plank	Time held: 30 s – 60 s R & L 1–2 reps Performed with one leg lifted	Time held: 30 s – 60 s R & L 2–3 reps Performed with one leg lifted	Time held: 30 s – 60 s 2–3 reps Performed with feet wide and one arm across chest
Wall slides (with exercise ball)	15–20 reps 1–2 sets	20 reps 2–3 sets	25–30 reps 3 sets Dumbbells	Lunges	20 reps R & L 2–3 sets Dumbbells Static	20 reps R & L 2–3 sets Front lunge Dumbbells	30 reps R & L 3 sets Walking lunge Dumbbells
-	-	-	-	Crunches on exercise ball	12–15 reps 2 sets	15 reps 2–3 sets	10–12 reps 2–3 sets Reverse crunches (feet on ball)
**Stretches**							
Doorway chest stretch	Time held: 20 s (R & L) 2 sets	Time held: 20 s (R & L) 3–4 sets	Time held: 30 s (R & L) 3–4 sets	Doorway chest stretch/side lunge stretch	Time held: 20 s (R & L) 2 sets	Time held: 20 s (R & L) 3–4 sets	Time held: 30 s (R & L) 3–4 sets
Neck stretches	Time held: 10 s each 2 sets	Time held: 10 s each 3–4 sets	Time held: 10 s each 3–4 sets	Neck stretches	Time held: 20 s each 3–4 sets	Time held: 20 s each 3–4 sets	Time held: 20 s each 3–4 sets
Lying on back, knees side to side (low back)	15–20 reps 1–2 sets	20 reps 1–2 sets	25–30 reps 1–2 sets	Lying on back, knees side to side (low back) with feet off ground	10–12 reps 1–2 sets	12–15 reps 2 sets	12–15 reps 2–3 sets
Hamstring stretch with towel	Time held: 20 s (R & L) 2 sets	Time held: 20 s (R & L) 3–4 sets	Time held: 30 s (R & L) 3–4 sets	Hamstring stretch with towel	Time held: 30 s (R & L) 3–4 sets	20 reps (R & L) 3 sets Dorsiflexion /plantarflexion of ankle	30 reps (R & L) 3 sets Dorsiflexion /plantarflexion of ankle
Abdominal and chest breathing	10 reps each 1 set	15 reps each 1 set	15 reps each 2 sets	Bellows breathing, diaphragmatic breathing	15 s – 30 s 2 min	30 s – 60 s 2 min	30 s – 60 s 2 min
Lying on back knee to chest stretch (hip flexors)	Time held: 20 s (R & L) 2 sets	Time held: 20 s (R & L) 3–4 sets	Time held: 30 s (R & L) 3–4 sets	Lying on back knee to chest stretch (hip flexors)	Time held: 20 s (R & L) 2 sets	Time held: 20 s (R & L) 2 sets	Time held: 30 s (R & L) 2 sets
-	-	-	-	Lying on back knee to side (low back). Opposite hand to knee	Time held: 20 s (R & L) 2 sets	Time held: 20 s (R & L) 2 sets	Time held: 30 s (R & L) 2 sets
-	-	-	-	Downward dog into upward dog	Time held: 20 s 2 sets	Time held: 20 s 2 sets	Time held: 30 s 2 sets

L, left; min, minutes; reps, repetitions; R, right; s, seconds.

## Statistical analysis

International Business Machines Corporation Statistical Package for the Social Sciences Statistics 19 was used for the statistical analysis. As a result of the small sample size, non-parametric Wilcoxon signed ranks tests, which are analogous to paired sample *t*-tests, were conducted, and the medians are reported. Mann–Whitney U non-parametric tests, analogous to independent *t*-tests for two samples, were used to compare the two groups before the onset of our study to determine whether any one group differs from the other with regard to any of the important covariates. Post-intervention Mann–Whitney U-tests were also performed to compare the two groups after the 6-month intervention. These tests were based on the available sample sizes at that time. All reported *p*-values are two-tailed. Effect sizes (ES) are reported for the between group comparisons on results that indicated substantial differences between the two groups (*p* = 0.05). Cohen (1992) suggested *r* = 0.1 constitutes a small effect size, *r* = 0.3 a moderate effect size and *r* = 0.5 a large effect size.

### Ethical considerations

Ethical approval for the study was obtained from the Postgraduate and Ethics Committee of the University of Pretoria (Faculty of Humanities) on 29 February 2012, no clearance numbers were assigned at the time.

## Results

A total of 29 (14 females, 15 males) human leukocyte antigen-B27 positive patients who were diagnosed according to the Modified New York Criteria participated in our study. A description of the participants per group (ASE and ASC groups) is presented (Table 2). Large *p*-values (between 0.09 and 1) confirmed that it is unlikely that the two groups differ with respect to their demographic characteristics. Four participants in the ASE group and five in the ASC group did not complete the exercise programme. The final numbers were *n* = 12 (six males and six females) for the ASE group and *n* = 8 (three males and five females) in the ASC group. The ASE group had a mean age (years) of (mean ± SD) 42 ± 15, range (21–66) and the ASC group (mean ± SD) 42 ± 11, range (28–63). There were various reasons for some of participants not completing the exercise programme. For the ASC group reasons included: time constraints, difficulty travelling for the assessments and work commitments. For the ASE group reasons included: time constraints, difficulty travelling and the motivation to continue with the exercise programme over a relatively long period of time. Only the data of participants who completed the programme were included in the statistical analysis.

**TABLE 2:** Description of the participants (*n* = 29).

Variable	An exercise group		A control group	
	*n* = 16	Mean ± SD	*n* = 13	Mean ± SD
Age (years)	39.44	14.8	39.00	12.8
Males (*n*)	9	-	6	-
Females (*n*)	7	-	7	-
Body mass (kg)	77.4	18.3	81.1	21.5
Stature (cm)	173.3	13.0	174.2	8.8
Body mass index (BMI)	25.7	5.5	26.7	5.4

### Between group analyses

The two groups were comparable concerning all blood, physical and functional assessments before the onset of the intervention (Table 3 – Table 5). The blood assessments including ESR (*p* = 0.562) and CRP (*p* = 0.35) did not show appreciable changes between the groups after the intervention (Table 3). The physical assessments favoured the ASE group with evidence of the incompatibility of the data with the null hypothesis of no improvements in CWE (*p* = 0.002), FEV1% (*p* = 0.012), absolute and relative VO_2max_ (*p* = 0.017; *p* = 0.003) as well as absolute and relative PWC (*p* = 0.028; *p* = 0.001) as presented (Table 4). The ES all indicate a large effect. Comparing the post-intervention functional assessments of the groups (Table 5) demonstrates that the ASE group improved in all variables, compared with the ASC group (BASFI, *p* = 0.007; BASDAI, *p* = 0.002; BASMI, *p* = 0.003), with large ES.

**TABLE 3:** Blood assessments pre-and post-intervention, within and between groups (*n* = 20).

Variable	An exercise group (*n* = 12) (Median)				A control group (*n* = 8) (Median)				Between group comparisons (*p*)	
	Pre-intervention	Post-intervention	*p*	↑/↓	Pre-intervention	Post-intervention	*p*	↑/↓	Pre-intervention	Post-intervention
ESR (mm/hr)	2.00	2.00	0.707	=	3.50	2.00	1.000	↓	0.936	0.562
CRP (µg/mL)	4.85	2.55	0.253	↓	3.00	6.00	0.103	↑	0.579	0.350

CRP, C-reactive protein; ESR, erythrocyte sedimentation rate; *n*, post-intervention number.

**TABLE 4:** Physical assessments pre-and post-intervention, within and between groups (*n* = 20).

Variable	An exercise group (*n* = 12) (Median)				A control group (*n* = 8) (Median)				Between group comparisons *p* (Effect size)	
	Pre-intervention	Post-intervention	*p*	↑/↓	Pre-intervention	Post-intervention	*p*	↑/↓	Pre-intervention	Post-intervention
BM	77.80	76.10	0.525	↓	75.10	74.05	0.472	↓	0.475	0.970
BMI	25.60	24.70	0.597	↓	25.55	25.25	0.374	↓	0.503	0.792
PBF	23.80	22.70	0.001*	↓	30.75	32.00	0.410	↑	0.092	0.098
LBM	53.80	54.50	0.019*	↑	54.65	52.55	0.303	↓	0.880	0.624
Endomorphy	5.50	4.45	0.001*	↓	5.80	6.00	0.733	↑	0.083	0.082
Mesomorphy	4.90	5.30	0.001*	↑	4.50	4.40	0.004*	↓	0.424	0.471
Ectomorphy	1.40	1.65	0.460	↑	1.25	1.20	0.367	↓	0.589	0.678
CWE	3.00	5.15	0.008*	↑	4.05	3.10	0.023*	↓	0.423	0.002*
										(0.66)**
FEV1 (mL)	3.10	3.12	0.940	↑	2.93	2.97	0.673	↑	0.440	0.518
FEV1%	102.85	109.00	0.124	↑	88.10	79.00	0.173	↓	0.254	0.012*
										(0.55)**
FVC (mL)	3.69	4.00	0.657	↑	3.64	4.17	0.963	↑	0.496	0.699
FEV1/FVC (%)	100.50	96.50	0.129	↓	94.00	91.00	0.673	↓	1.000	0.518
PEF (L/min)	7.72	8.03	0.291	↑	7.44	7.28	1.000	↓	0.683	0.518
VO_2max_ absolute (L/min)	2.45	3.55	0.001*	↑	2.60	2.30	0.024*	↓	0.486	0.017*
										(0.53)**
VO_2max_ relative (mL/kg/min)	32.25	43.10	0.001*	↑	32.50	25.30	0.073	↓	0.249	0.003*
										(0.64)**
PWC absolute (w)	107.00	145.00	0.001*	↑	130.00	115.00	0.037*	↓	0.682	0.028*
										(0.49)**
PWC relative (w/kg)	1.40	1.90	0.001*	↑	1.30	1.10	0.004*	↓	0.475	0.001*
										(0.68)**
BOS right	2.45	1.75	0.001*	↓	3.10	3.00	0.016*	↓	0.170	0.082
BOS left	2.95	1.70	0.014*	↓	2.30	2.50	0.964	↑	0.812	0.167

BMI, body mass index; BM, body mass; BOS, balance overall stability; CWE, chest wall expansion; FEV1, forced expiration value of the first second; FEV1%, % of predicted FEV1; FVC, forced vital capacity; LBM, lean body mass; PBF, percentage body fat; PEF, peak expiratory flow; PWC, physical work capacity; VO_2max_, maximal oxygen consumption; *n*, post-intervention number; ↑/↓, direction of change.

* , *p* < 0.05.

** , effect size.

**TABLE 5:** Functional assessments pre-and post-intervention, within and between groups (*n* = 20).

Variable	An exercise group (*n* = 12) (Median)				A control group (*n*= 8) (Median)				Between group comparisons *p* (Effect size)	
	Pre-intervention	Post-intervention	*p*	↑/↓	Pre-intervention	Post-intervention	*p*	↑/↓	Pre-intervention	Post-intervention
BASFI	2.48	0.95	0.798	↓	4.18	4.7	0.848	↑	0.056	0.007*
										(0.59)**
BASDAI	4.04	1.56	0.032*	↓	5.89	5.15	1.000	↓	0.779	0.002*
										(0.67)**
BASMI	1.8	0.70	< 0.001*	↓	1.5	1.9	0.081	↑	0.232	0.003*
										(0.63)**

BASDAI, bath ankylosing spondylitis disease index; BASFI, bath ankylosing spondylitis functional index; BASMI, bath ankylosing spondylitis metrology index; *n*, post-intervention number; ↑/↓, direction of change.

* , *p* < 0.05.

** , Effect size.

### Within group analyses

Within both groups, the inflammatory blood markers as measured by ESR and CRP did not change statistically (*p*-ranges between 0.103 and 1, Table 3). The ASE group had statistical improvements in 11 of the 20 physical variables assessed as presented (Table 4). These included: PBF decreased (*p* = 0.001), LBM increased (*p* = 0.019), endomorphy decreased (*p* = 0.001), mesomorphy increased (*p* = 0.001), CWE increased (*p* = 0.008), absolute and relative VO_2max_ increased (*p* = 0.001; *p* = 0.001), absolute and relative PWC increased (*p* = 0.001; *p* = 0.001) and balance overall stability (BOS) improved on the right (*p* = 0.001) and on the left (*p* = 0.014). In the ASC, there was statistical evidence of deterioration in five of the physical variables assessed, including mesomorphy (*p* = 0.004), CWE (*p* = 0.023), absolute VO_2max_ (*p* = 0.024) and absolute and relative PWC (*p* = 0.037; *p* = 0.004) as shown (Table 4). The ASC group showed improvement only in BOS on the right (*p* = 0.016). The functional assessment results (Table 5) for the ASE group post-intervention had evidence of improvements in the BASDAI (*p* = 0.032) and BASMI (*p* = 0.001) whilst the ASC group remained unchanged.

## Discussion

Our study is one of few that has been carried out over a 6-month period, and consisted of a combination of exercises (land, swimming and breathing exercises) and involved a comprehensive assessment that included pulmonary function and balance (stability). The main findings of the effect of a 6-month exercise intervention in AS patients in this randomised control trial include: (1) no disease exacerbation with improved subjective (BASFI, BASDAI) and objective (CRP, BASMI) clinical parameters, (2) improvement of physical function, (3) improved chest expansion, (4) increased aerobic capacity and (5) better postural stability.

The first important finding was that the exercise intervention did not exacerbate the disease activity of the AS participants when considering the ESR and CRP. There was a clinical improvement within the ASE group’s CRP value; however, the improvement was not statistically significant. Furthermore, the ASE group compared with the ASC group had better quality of life, fewer symptoms and improved spinal flexibility, confirming results from other studies (Dundar et al. 2014). Therefore, it appears as if the combined programme of swimming and land-based exercises can be performed without the fear of worsening disease activity and even lead to reduced disease activity and better quality of life as was shown in a previous study by our group where exercise leads to disease remission in rheumatoid arthritis (RA) patients (Janse van Rensburg et al. 2012).

The second important finding is that there was improvement in 11 of the physical assessment scores within the ASE group. The exercise intervention appears to have had a positive effect on the AS participants’ anthropometric measurements and body composition. The ASE group had improvements in PBF, LBM and the endomorphy and ectomorphy components. This positive effect is very important as many AS patients tend to be sedentary and studies have shown that AS is associated with increased cardiovascular morbidity (Haroon et al. 2015). Therefore, the benefits of exercise such as improved body composition can possibly prevent or assist in the treatment of comorbid chronic conditions. Ankylosing spondylitis patients may have diseases such as Type II diabetes or hypertension. The general trend of the anthropometry and body composition scores within the ASC group was that of deterioration; however, statistically there were no differences between the ASE and ASC groups.

It has been shown that pulmonary involvement is common in patients with AS (Sampaio-Barros et al. 2007). Rigidity of the thorax occurs in AS with bony ankyloses of the thoracic vertebrae, costovertebral, costotransverse, sternoclavicular and sternomanubrial joints (Fisher, Cawley & Holgate 1990). The third important finding of our 6-month exercise intervention is that this combination of exercises appears to be effective in improving thoracic expansibility in AS patients. The CWE improved within the ASE group whilst there was a deterioration within the ASC group. As a result, the CWE was statistically different between the two groups post-intervention. The role of respiratory exercises in AS has been supported by previous studies (Daginfrud et al. 2011; Soloman et al. 1975; Drӑgoi et al. 2016; So et al. 2012). Pulmonary function tests in AS have revealed a high prevalence of restrictive defects, characterised by a low FVC (Sampaio-Barros et al. 2007). The general trend of the pulmonary function values within the ASE group was that of small improvements. The results were inconsistent for the ASC group, all the pulmonary function values within this group decreased slightly except for FVC. The FEV1% was statistically higher in the ASE group compared with the ASC group post-intervention. Studies evaluating the effects of exercise on pulmonary functions are limited. Durmus et al. (2009) found statistically significant improvements in chest expansion and pulmonary function parameters such as FVC and FEV1 in AS patients following home-based exercises (Durmus et al. 2009). Ince et al. (2006) found a 3-month exercise intervention (multimodal exercise programme) also significantly increased chest expansion and FVC in AS patients (Ince et al. 2006). The results of our study are similar to those of Josenhans et al. (1971), who studied the effects of physiotherapy in 222 AS patients and found that pulmonary function parameters remained mostly unchanged despite improvements in chest wall and spinal mobility (Josenhans et al. 1971).

Aerobic exercises such as swimming or walking are often recommended in addition to conventional exercises for patients with AS (Elyan & Khan 2008). A decrease in functional capacity can often be observed in patients with AS because of musculoskeletal or pulmonary impairment (Fisher et al. 1990). It has also been shown that there is an inverse relationship between cardiorespiratory fitness measured from an exercise test and the risk of mortality (Arena et al. 2007). In addition, recent studies have revealed that AS is also associated with an increased risk of cardiovascular morbidity (Haroon et al. 2015). Therefore, it is important that exercise interventions for patients with AS target other aspects of physical fitness (Dagfinrud et al. 2011). Considering the importance of aerobic capacity, the positive influence of the 6-month exercise programme on the VO_2max_ and PWC as the fourth finding is promising and emphasises the importance of exercising for patients with AS. Especially, as there was a substantial deterioration in the ASC group in their absolute VO_2max_ and PWC (absolute and relative) over a relatively short period of time and that the ASE group’s aerobic capacity measures were higher compared with the ASC group at the end of our study. Other studies have shown similar improvements in aerobic capacity (Sveaas et al. 2014).

The postural changes characteristic of AS can result in changes in gait and balance impairment. These changes could lead to increased risk of falling (Zão & Cantista 2017). Limited studies have evaluated the effect of specific programmes on strength, cardiorespiratory and functional factors such as balance (Millner et al. 2016). The fifth finding of our study was that the ASE group had a notable improvement in overall stability (right and left) as measured by the Biodex balance SD^TM^. Although the ASC group had similar findings in overall stability on the right side, this may be because of repetition as a result of the testing that could have led to improvements. Parraca et al. (2011) found the Biodex balance SD^TM^ to be a reliable and a useful measure of balance and risk of falls. Therefore, further research regarding the effect of exercise interventions on balance in AS patients is required (Parraca et al. 2011).

Poor compliance and incorrect understanding on how to perform exercises are possible explanations of why exercise programmes fail. Also, the evidence supports the findings that supervised group exercise programmes are more effective than home exercise programmes (Cagliyan et al. 2007). However, studies comparing supervised- and home-exercise programmes are limited. In our study, participants in the exercise group each received an individual session with a biokineticist who went through the exercise programme to ensure that the participant understood and could perform the exercises properly. This could have contributed to the effectiveness of the exercise intervention. Furthermore, the ASE group kept an exercise log to determine compliance with the exercise programme. Compliance was acceptable as the AS participants attended 66.85% of their training sessions. Our study therefore supports the use of unsupervised programmes if supervision is not possible.

The limitations of our study include the small sample size of the two groups and the high drop-out rate. Larger samples will have more statistical power and allow parametric tests, which are more sensitive to detect changes, should there be any. Group allocation was not blinded for the assessor with a potential risk of bias. In addition, the same biokineticist who did the assessments conducted the intervention, which also may have biased the results. The wide age range and the groups containing both males and females could have also negatively influenced the results.

## Conclusions

The main findings of our study show that the exercise intervention was effective in improving clinical parameters and physical and functional capacity without exacerbating the disease status of the participants. Therefore, it appears as if an unsupervised, well explained exercise programme combining swimming exercises, land-based strengthening and stretching exercises and breathing exercises may be a good option for patients with AS. These findings must, however, be considered within the light of the limitations and therefore should be interpreted with caution.

## References

[CIT0001] Arena, R., Myers, J., Williams, M.A., Gulati, M., Kligfield, P., Balady, G.J. et al., 2007, ‘Assessment of functional capacity in clinical and research settings: A scientific statement from the american heart association committee on exercise, rehabilitation, and prevention of the council on clinical cardiology and the council on cardiovascular nursing’, *Circulation* 116(3), 329–343. 10.1161/circulationaha.106.184461 17576872

[CIT0002] Cagliyan, A., Kotevoglu, N., Onal, T. & Tekkus, B.B.K., 2007, ‘Does group exercise program add anything more to patients with ankylosing spondylitis?’, *Journal of Back & Musculoskeletal Rehabilitation* 20(2–3), 79–85. 10.3233/BMR-2007-202-305

[CIT0003] Cohen, J., 1992, ‘A power primer’, *Psychological Bulletin* 112(2), 155–159. 10.1037/0033-2909.112.1.155 19565683

[CIT0004] Dagfinrud, H., Halvorsen, S., Vøllestad, N.K., Niedermann, K., Kvien, T.K. & Hagen, K.B., 2011, ‘Exercise programs in trials for patients with ankylosing spondylitis: Do they really have the potential for effectiveness?’, *Arthritis Care & Research (Hoboken)* 63(4), 597–603. 10.1002/acr.20415 21452270

[CIT0005] Dean, L.E., MacDonald, A.G., Downham, C., Sturrock, R.D. & Macforlane, GJ., 2014, ‘Global prevalence of Ankylosing Spondylitis’, *Rhematology* 53(4), 650–657. 10.1093/rheumatology/ket387 24324212

[CIT0006] Dougados, M. & Van der Heijde, D., 2002, ‘Ankylosing spondylitis: How should the disease be assessed?’, *Best Practice & Research: Clinical Rheumatology* 16(4), 605–618. 10.1053/berh.2002.0252 12406429

[CIT0007] Drăgoi, R.G., Amaricai, E., Drăgoi, M., Popoviciu, H. & Avram, C., 2016, ‘Inspiratory muscle training improves aerobic capacity and pulmonary function in patients with ankylosing spondylitis: A randomised controlled study’, *Clinical Rehabilitation* 30(4), 340–346. 10.1177/0269215515578292 25810425

[CIT0008] Dundar, U., Solak, O., Toktas, H., Demirdal, U.S., Subasi, V., Kavuncu, V. et al., 2014, ‘Effect of aquatic exercise on ankylosing spondylitis: A randomised controlled trial’, *Rheumatology International* 34(11), 1505–1511. 10.1007/s00296-014-2980-8 24626605

[CIT0009] Durmuş, D., Alayli, G., Uzun, O., Tander, B., Cantürk, F., Bek, Y. et al., 2009, ‘Effects of two exercise interventions on pulmonary functions in the patients with ankylosing spondylitis’, *Joint Bone Spine* 76(2), 150–155. 10.1016/j.jbspin.2008.06.013 19084457

[CIT0010] Elyan, M. & Khan, M.A., 2008, ‘Does physical therapy still have a place in the treatment of ankylosing spondylitis?’, *Current Opinion in Rheumatology* 20(3), 282–286. 10.1097/BOR.0b013e3282fa13c9 18388519

[CIT0011] Fernández García, R., Sánchez-Sánchez, L. de.C., López Rodríguez, M. de.M. & Sánchez Granados, G., 2015, ‘Effects of an exercise and relaxation aquatic program in patients with spondyloarthritis: A randomised trial’, *Medicina Clinica (Barc)* 145(9), 380–384. 10.1016/j.medcli.2014.10.015 25639496

[CIT0012] Fisher, L.R., Cawley, M.I. & Holgate, S.T., 1990, ‘Relation between chest expansion, pulmonary function, and exercise tolerance in patients with ankylosing spondylitis’, *Annals of the Rheumatic Diseases* 49(11), 921–925. 10.1136/ard.49.11.921 2256739 PMC1004263

[CIT0013] Haroon, N.N., Paterson, J.M., Li, P., Inman, R.D. & Haroon, N., 2015, ‘Patients with ankylosing spondylitis have increased cardiovascular and cerebrovascular mortality: A population-based study’, *Annuals of Internal Medicine* 163(6), 409–416. 10.7326/m14-2470 26258401

[CIT0014] Heikkilä, S., Viitanen, J.V., Kautiainen, H. & Kauppi, M., 2000, ‘Sensitivity to change of mobility tests: Effect of short term intensive physiotherapy and exercise on spinal, hip, and shoulder measurements in spondyloarthropathy’, *Journal of Rheumatology* 27(5), 1251–1256. 10813296

[CIT0015] Hidding, A., Van der Linden, S., Boers, M., Gielen, X., De Witte, L., Kester, A. et al., 1993, ‘Is group physical therapy superior to individualized therapy in ankylosing spondylitis? A randomised controlled trial’, *Arthritis Care & Research* 6(3), 117–125. 10.1002/art.1790060303 8130287

[CIT0016] Hu, X.M., Chen, J.L., Tang, W.J., Chen, W.J., Sang, Y. & Jia, L.N., 2020, ‘Effects of exercise programmes on pain, disease activity and function in ankylosing spondylitis: A meta-analysis of randomised controlled trials’, *European Journal of Clinical Investigation* 50(12), 1–10. 10.1111/eci.13352 32683694

[CIT0017] Ince, G., Sarpel, T. & Durgun, B.S.E., 2006, ‘Effects of a multimodal exercise program for people with ankylosing spondylitis’, *Physical Therapy* 86(7), 924–935. 10.1093/ptj/86.7.924 16813473

[CIT0018] Janse van Rensburg, D.C., Ker, J.A., Grant, C.C. & Fletcher, L., 2012, ‘Effect of exercise on cardiac autonomic function in females with rheumatoid arthritis’, *Clinical Rheumatology* 31(8), 1155–1162. 10.1007/s10067-012-1985-5 22526478

[CIT0019] Josenhans, W.T., Wang, C.S., Josenhans, G. & Woodbury, J.F., 1971, ‘Diaphragmatic contribution to ventilation in patients with ankylosing spondylitis’, *Respiration* 28(4), 331–346. 10.1159/000192822 5138483

[CIT0020] Karapolat, H., Eyigor, S., Zoghi, M., Akkoc, Y., Kirazli, Y. & Keser, G., 2009, ‘Are swimming or aerobic exercise better than conventional exercise in ankylosing spondylitis patients? A randomised controlled study’, *European Journal of Physical Rehabilitation Medicine* 45(4), 449–457. 20032902

[CIT0021] Karatepe, A.G., Akkoc, Y., Akar, S., Kirazli, Y. & Akkoc, N., 2005, ‘The Turkish versions of the bath ankylosing spondylitis and dougados functional indices: Reliability and validity’, *Rheumatology International* 25(8), 612–618. 10.1007/s00296-004-0481-x 15248085

[CIT0022] Lim, H.J., Lim, H.S. & Lee, M.S., 2005, ‘Relationship between self-efficacy and exercise duration in patients with ankylosing spondylitis’, *Clinical Rheumatology* 24(4), 442–443. 10.1007/s10067-004-0974-8 15338448

[CIT0023] Marfell-Jones, M. & Olds, T., 2006, *International standards for anthropometric assessment*, The International Society for Advancement in Kinanthropometry, Canberra.

[CIT0024] Miller, M.R., Hankinson, V., Brusasco, V., Burgos, F., Casaburi, R., Coates, A. et al., 2005, ‘Series: “ATS/ERS task force: Standardisation of lung function testing”’, *European Respiratory Journal* 26, 319–338. 10.1183/0903136.05.00034805 16055882

[CIT0025] Millner, J.R., Barron, J.S., Beinke, K.M., Butterworth, R.H., Chasle, B.E., Dutton, L.J. et al., 2016, ‘Exercise for ankylosing spondylitis: An evidence-based consensus statement’, *Seminars Arthritis and Rheumatism* 45(4), 411–427. 10.1016/j.semarthrit.2015.08.003 26493464

[CIT0026] Nolte, K. & Janse van Rensburg, D.C., 2001, ‘The role of exercise in the rehabilitation of ankylosing spondylitis’, *International Sports Medicine Journal* 2(4), 1–12.

[CIT0027] O’Dwyer, T., O’Shea, F. & Wilson, F., 2014, ‘Exercise therapy for spondyloarthritis: A systematic review’, *Rheumatology International* 34(7), 887–902. 10.1007/s00296-014-2965-7 24549404

[CIT0028] Özdemir, O., 2011, ‘Quality of life in patients with ankylosing spondylitis: Relationships with spinal mobility, disease activity and functional status’, *Rheumatology International* 31(5), 605–660. 10.1007/s00296-009-1328-2 20049451

[CIT0029] Parraca, J.A., Olivares, P.R., Carbonell-Baeza, A., Aparicio, V.A., Adsuar, J.C. & Gusi, N., 2011,‘Test-retest reliability of Biodex balance sd on physically active old people’, *Journal of Human Sport & Exercise* 6(2), 444–451. 10.4100/jhse.2011.62.25

[CIT0030] Sampaio-Barros, P.D., Cerqueira, E.M., Rezende, S.M., Maeda, L., Conde, R.A., Zanardi, V.A. et al., 2007, ‘Pulmonary involvement in ankylosing spondylitis’, *Clinical Rheumatology* 26(2), 225–230. 10.1007/s10067-006-0286-2 16572281

[CIT0031] So, M.W., Heo, H.M., Koo, B.S., Kim, Y.G., Lee, C.K. & Yoo, B., 2012, ‘Efficacy of incentive spirometer exercise on pulmonary functions of patients with ankylosing spondylitis stabilized by tumor necrosis factor inhibitor therapy’, *Journal of Rheumatology* 39(9), 1854–1858. 10.3899/jrheum.120137 22859343

[CIT0032] Soloman, L., Beighton, P., Valkenburg, H.A., Robin, G. & Soskoline, C.L., 1975, ‘Rheumatic disorders in the South African Negro: Part 1: Rheumatoid Arthritis and Ankylosing Spondylitis’, *SA Medical Journal* 49, 1292–1296. 1154196

[CIT0033] Sveaas, S.H., Berg, I.J., Provan, S.A., Semb, A.G., Hagen, K.B., Vøllestad, N. et al., 2014, ‘Efficacy of high intensity exercise on disease activity and cardiovascular risk in active axial spondyloarthritis: A randomised controlled pilot study’, *PLoS One* 9(9), e108688. 10.1371/journal.pone.0108688 25268365 PMC4182541

[CIT0034] Ward, M.M., Weisman, M.H., Davis, J.C. & Reveille, Jr. J.D., 2005, ‘Risk factors for functional limitations in patients with long-standing ankylosing spondylitis’, *Arthritis & Rheumatology* 53(5), 710–717. 10.1002/art.21444 PMC2530902 16208654

[CIT0035] Zão, A. & Cantista, P., 2017, ‘The role of land and aquatic exercise in ankylosing spondylitis: A systematic review’, *Rheumatology International* 37(12), 1979–1990. 10.1007/s00296-017-3829-8 28983663

